# Understanding Nurses’ Views on the Principles and Exercise of Family-Centered Care: A Qualitative Exploration

**DOI:** 10.7759/cureus.105806

**Published:** 2026-03-24

**Authors:** Alfianoora Kabeer Nusaifa, Hepsi Bai Joseph

**Affiliations:** 1 Nursing, Saint Marys Hospital, Manchester, GBR; 2 College of Nursing, All India Institute of Medical Sciences, Bhubaneswar, Bhubaneswar, IND

**Keywords:** family-centered care, nurses, perception, principles, qualitative study

## Abstract

Introduction: Family-centered care (FCC) is a central element of child health nursing. It describes a way of caring for sick children and their families within the health service, consistently ensuring that care is planned for the whole family rather than just for the child. The present study aimed to explore the perception of nurses on FCC.

Methods: A qualitative explorative design was used to inquire into the perception of FCC among nurses from different pediatric units. Three focus group discussions were conducted among nurses to obtain detailed information on the topic. The transcribed verbatim was analyzed using conventional thematic analysis, and data management was performed using NVivo software (Lumivero, Denver, CO, USA).

Results: Data were analyzed using a systematic thematic analysis approach. Related codes were compared and clustered into subthemes. Themes were then developed by synthesizing related subthemes into higher-level interpretive constructs. Two major themes emerged from the nurses’ verbatim accounts: “Grounds of Family-Centered Care (FCC)” and “FCC as a Comprehensive Process of Child Care.” The theme *Grounds of FCC* comprised subthemes related to parental factors and child-related factors that influence the implementation of FCC. The second theme, *FCC as a Comprehensive Process of Child Care*, included the subthemes child-centred care, family-centred care, and nurse-centred care, reflecting the multidimensional nature of FCC in clinical practice.

Conclusion: The study highlights that pediatric nurses perceive FCC as a fundamental and comprehensive approach to child health nursing. Nurses emphasized that FCC is grounded in parental and child-related factors and is integral to delivering holistic care. The findings further reveal that FCC is understood as a dynamic process encompassing child-centred, family-centred, and nurse-centred dimensions. Strengthening nurses’ understanding and support of FCC principles may enhance collaborative care practices and improve outcomes for children and their families.

## Introduction

Children constitute a vital population group whose health and developmental outcomes have profound implications for national productivity, social stability, and intergenerational equity, as emphasized in international child health and development frameworks. Healthy children develop into healthy adults. Hence, the proper physical and emotional growth and development of a child play a crucial role in producing a healthy citizen [1]. Empirical investigations suggest that during hospitalisation, children display anxiety through regression in behaviours, anger, disobedience, withdrawal, and difficulties in recovering from procedures [2]. Regardless of the child’s illness, the child’s hospitalisation can be a traumatic experience for the parents and the entire family. Adaptation to the new environment and parental responsibilities is essential during this stage. The presence of a family member during treatment can significantly reduce both the child’s and the parent’s anxiety. Staying together with the child can also help the parents understand the disease and prepare them for post-discharge care [3]. In family-centered care (FCC), the health care provider recognises and utilises the families’ knowledge of their family member’s condition and the families’ ability to communicate with their family members [4]. The entire family becomes the unit of care, which means that carers are focused on the admitted child and the rest of the family. Nurses’ perceptions of the family’s participation in the hospitalized child’s care, nurses’ relationships with parents, communication channels and styles, environmental constraints, and staff management support are all elements that influence the successful implementation of FCC [5]. Paediatric nurses have a unique opportunity to help families during a challenging time by facilitating parental participation in child care. However, with growing consumer demands, nurses face difficulty performing their roles [6]. Although a few studies have explored aspects of FCC in India, such as acceptability and feasibility of FCC models in neonatal intensive care settings, the overall body of research remains limited and largely context-specific. Additionally, while feasibility studies in neonatal units suggest potential benefits of FCC, there is a paucity of systematic research synthesizing FCC practices, perceptions, and barriers across diverse Indian healthcare settings, especially outside neonatal care. These gaps underscore the need for more contextually grounded studies that examine FCC from the perspectives of nurses and families across a wider range of paediatric care environments in India. There are pieces of literature evident on the FCC in the Western scenario, but not apparent in the Indian setting. Hence, the present study was undertaken to understand nurses’ perceptions of FCC.

## Materials and methods

Study design

A qualitative, exploratory, descriptive design was adopted to understand nurses’ perceptions and views of FCC.

Study setting

The study was conducted in selected paediatric units of the All India Institute of Medical Sciences (AIIMS), Bhubaneswar, an autonomous institute under the Ministry of Health and Family Welfare. AIIMS Bhubaneswar is a 978-bedded tertiary care hospital providing general and super-specialty services with round-the-clock emergency and critical care facilities. The paediatric inpatient services include the Paediatric Medicine ward (60 beds), Paediatric Surgery ward (32 beds), Neonatal Intensive Care Unit (NICU), and Paediatric Intensive Care Unit (PICU) (14 beds). For the present study, the Paediatric Medicine ward, Paediatric Surgery ward, and PICU were included. The NICU was excluded as FCC is already well established in this unit and has been extensively studied in the Indian context.

Participants and sampling

Paediatric nurses working in the selected units for at least two years and able to communicate in English or Hindi were included using purposive sampling. Nurses working at the managerial level, those on temporary reliever duty, and those unwilling to participate were excluded.

Focus group discussions

Three focus group discussions (FGDs) were conducted using a semi-structured, in-depth interview guide (Appendix) to explore nurses’ perceptions and practices related to FCC. The FGDs were organized according to unit availability to ensure representation from the Paediatric Medicine ward, Paediatric Surgery ward, and PICU. A total of 20 nurses participated across the three groups. The first FGD comprised seven participants, the second six, and the third seven. Each session lasted approximately 30-40 minutes and was conducted in a quiet, private room within the paediatric unit to ensure comfort and confidentiality. All sessions were moderated by the principal investigator, who has formal training and experience in qualitative research and focus group facilitation. The moderator encouraged open discussion, ensured equal participation, and used probing questions to elicit in-depth responses. A co-researcher was present during each session to take detailed field notes on non-verbal cues, group dynamics, and contextual observations, and to manage the audio recording. Immediately after each session, a brief debriefing was conducted between the moderator and co-researcher to document preliminary impressions and, where necessary, refine subsequent discussions.

Ethical considerations and data collection

Approval was obtained from the Institutional Ethics Committee of AIIMS (approval IEC/AIIMS BBSR/Nursing/2021-22/07) and permission from hospital authorities prior to data collection. Eligible nurses were provided with a Participant Information Sheet detailing the study purpose. Written informed consent was obtained before participation. Demographic data were collected prior to the interviews. Participants were informed about audio recording, and confidentiality was assured.

Data analysis and rigor

Data were analyzed using thematic analysis. Verbatim transcripts were prepared independently by the investigator and a non-nursing professional to ensure accuracy and congruence. As interviews were conducted in English, translation was not required. Manual line-by-line open coding generated codes related to both perception and practice. Analysis began after the first FGD and proceeded concurrently with data collection. Field notes documenting nurse-family interactions were incorporated into the analysis. Peer debriefing with the research guide and peer group was conducted after each session, and suggestions were integrated into subsequent interviews and coding. The report was prepared in accordance with the Standards for Reporting Qualitative Research (SRQR) checklist to ensure methodological rigor [7].

## Results

Demographic data were analysed using descriptive statistics and summarized as frequencies and percentages. Eighty percent of the participants’ age ranged from 26-35 years, and the majority (73.3%) of them were female. Regarding educational status, 60% of the nurses had a B.Sc. degree.

Views and perceptions of the FCC among nurses

**Figure 1:**
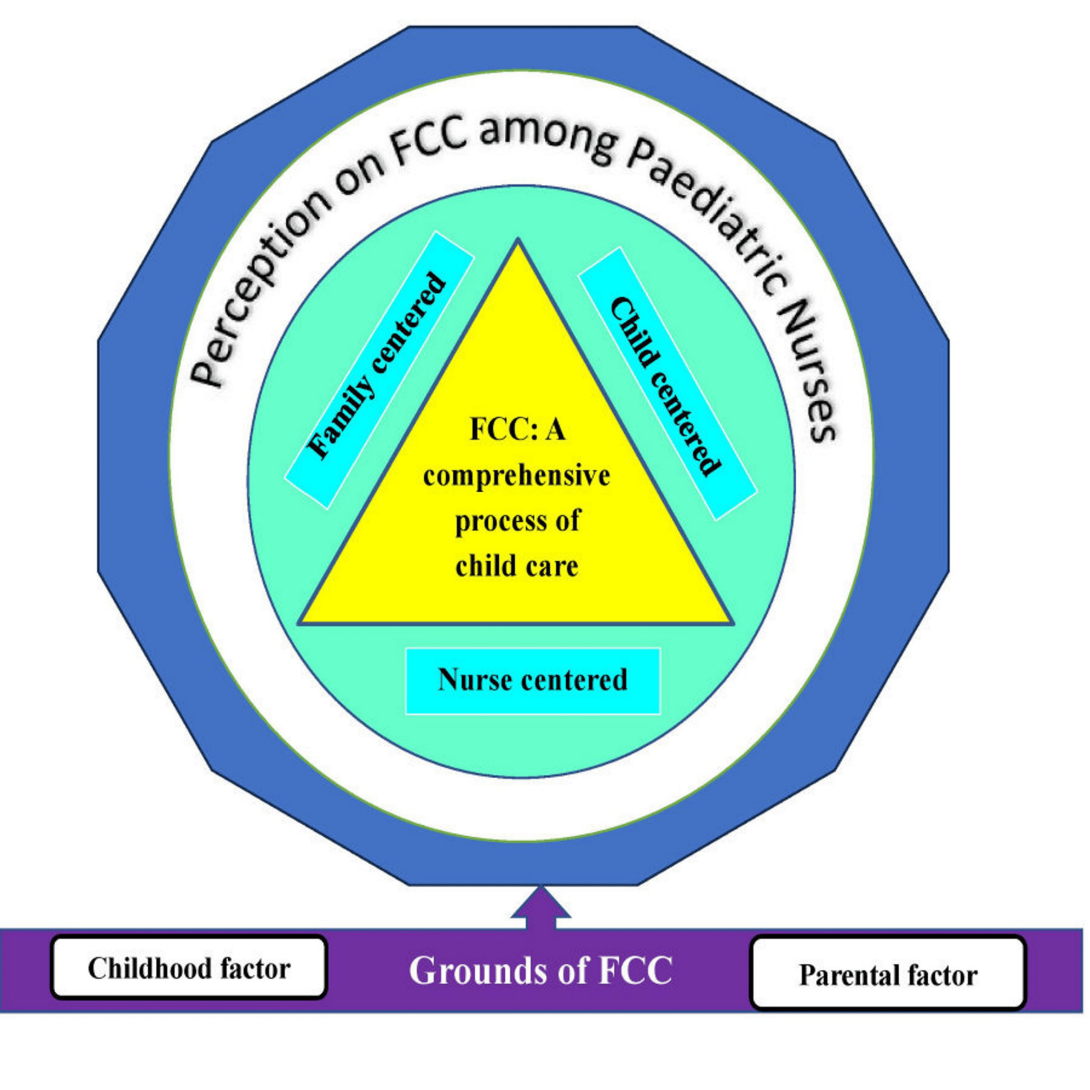
Conceptual diagram on perception of nurses on family-centered care (FCC) Image created by the authors using Microsoft Word (Microsoft Corporation, Redmond, WA, USA).

**Figure 2:**
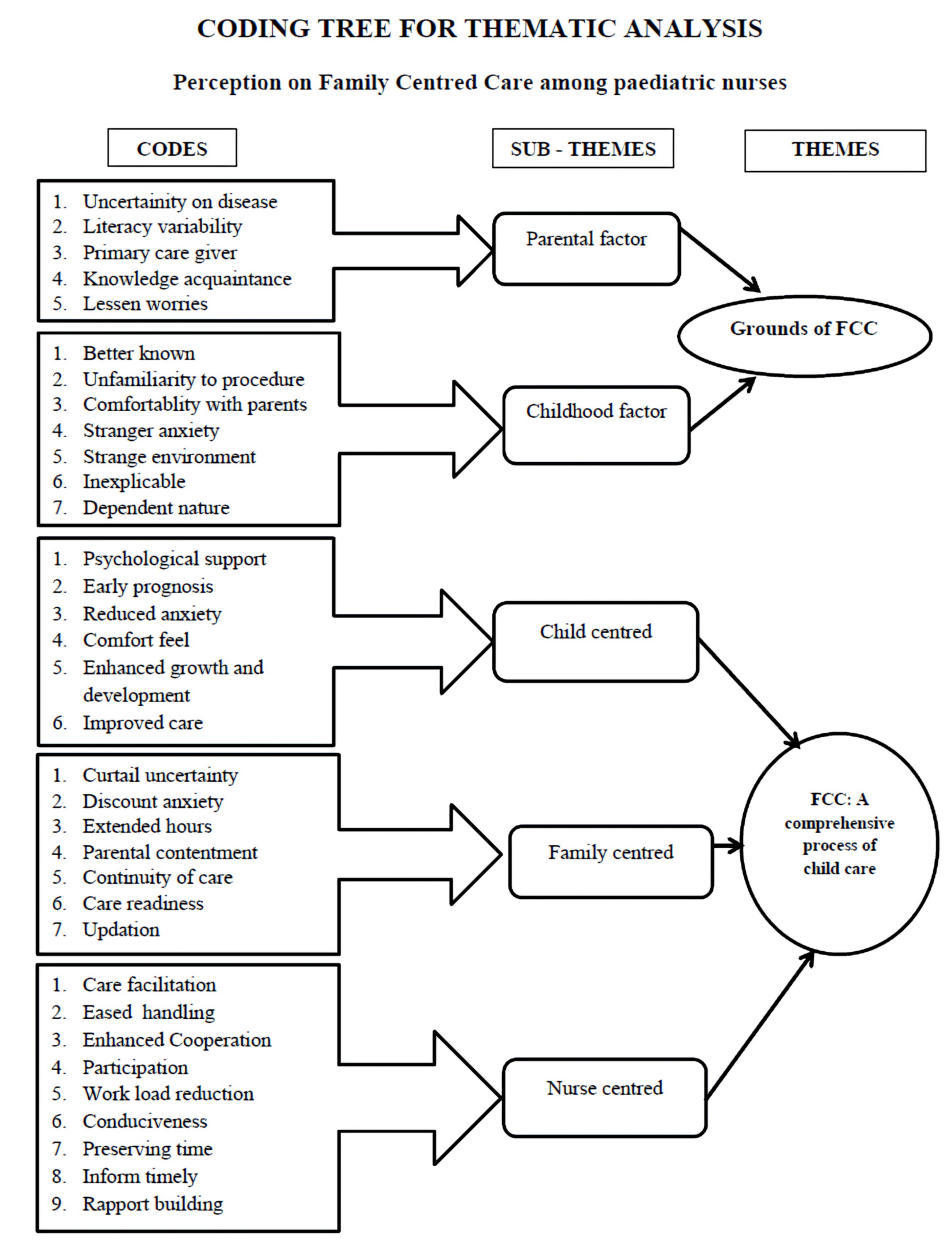
Themes and subthemes on perception on family-centered care (FCC) among nurses. Image created by the authors.

The findings were illustrated through an inductive process of data analysis. After coding the significant statements from the transcript of participants, the meaningful codes were condensed to subthemes and subsequently subthemes to themes. The findings are supported by the quotes from the participants. A conceptual framework depicting nurses’ perspectives and perceptions is presented in Figure 1. To ensure credibility, the interpretations are substantiated with direct quotations from participants. The coding tree of the analysis is presented in Figure 2.

Theme 1 - Grounds of FCC

Parental factor subtheme: The nurses expressed that parents are a significant factor for FCC. Parents were the primary caregivers who fulfilled all the child's basic needs. Without a parent’s presence, it is hard for children to survive in an unfamiliar environment. The paediatric nurses reported that when children were hospitalized, parents worried about the child's condition. Therefore, if parents were allowed to accompany their child in the hospital, it would help them understand the child's actual condition. Nurses had stated that even illiterate parents could understand their child’s disease when they were with the child.

“Actually, parents are the primary caregivers in paediatric care, first comes the parents, after that only all others.” (P15 PSW)

“I think it will be good for the child. Whenever the parents are coming, if they are doing anything to the baby under our supervision, we are teaching them. So it’s good for them”. (P3 PICU)

Childhood factor subtheme: The nurses addressed children as another essential factor for the need for FCC. They portrayed that the children were new to the hospital environment, and the procedures had increased their anxiety and discomfort. They stated that the presence of unfamiliar equipment and strangers makes the children worried and anxious.

“Children are afraid of nurses, the hospital environment, our hospital, and all. The procedure also seems to be painful for children. (P6 PMW.)

“Children will have stranger anxiety. Children are comfortable with their parents; otherwise, without parents, they are not comfortable, and they will not cooperate with us”. (P6 PMW)

Theme 2 - FCC: A Comprehensive Process of Child Care

Child-centered subtheme: Paediatric nurses depicted that hospitalized children need physical and emotional support to overcome the disease and promote their growth and development, even during the illness. Nurses shared their view that the FCC is child-centered, so the main focus of the FCC is providing optimum care to the child, as they are the center of care. Ultimately, children should receive care that promotes their physical, mental, and social health and well-being.

“In my opinion, if mother and father are there with the child, the child can recover fast from the disease they are suffering from because the hospital environment is made comfortable with parents only.” (P5 PMW)

“Family presence during hospitalization will be very beneficial for the child’s growth and development and maintenance of hygiene.” (P13 PMW)

Family-centered subtheme: As the parents and family members were adversely affected during their child’s hospitalization, nurses shared their outlook on family-centeredness as it reduced the parental worries and helped the parents to care for their child, to know in detail about the child’s condition and treatment. Nurses added that allowing parents or family members along with the child helped parents get more time to spend with their child, which indirectly helped to reduce their anxiety and achieve psychological satisfaction from doing something worthwhile for their child.

“When parents are involved in caring for the child, parents get more time to spend with their child.” (P13 PMW)

“It’s beneficial for the parents because, when the parents are staying along with the child, they can see the child’s progress directly, or whatever test is happening, or whatever is done to the child. They can know that and the actual clinical progress they can see”. (P8 PSW)

Nurse-centered subtheme: As part of comprehensive care, nurses could also involve the family in various aspects of child care and treatment, which helped them build a good rapport with the child and the family, thereby benefiting from the child’s cooperation and reducing their workload. The presence of family helped the nurses in various ways. The parental involvement in care activities helped them build an excellent interpersonal relationship with the child and the family, which in turn aided in understanding the family’s ability to care for the child.

“It’s easy to counsel the parents because, if any doctor or nurse wants to counsel or anything to the patient, it was easy to counsel if parents were there, and if we were giving any health education to them in hygiene, then they used to maintain that.” (P1 PMW)

“Parental presence will reduce our workload if we have two or three patients and we have to give NG tube feed at the same time. It will reduce our work stress”. (P3 PICU)

“The benefit is that time. We will get time to take care of other patients if parents are there”. (P13 PICU)

## Discussion

The current study explored nurses' perception of FCC. The interview provided the researcher with insight into nurses’ concepts of FCC. The study’s findings were similar to other published studies [8-10]. Even though nurses were not fully aware of the FCC as a whole, they were aware of its components and were practicing most of them in their settings. The pediatric nurses in this study acknowledged that parents play a pivotal role in children's lives, especially when they are ill. Consistent results reported in previous studies have demonstrated the value of parental presence and family continuity during a child’s hospitalization. Understanding the role of the family has led to the acceptance of the FCC concept [11]. As in other studies, nurses believed all elements of FCC were essential [12,13]. In previous studies on FCC, nurses opposed the constant presence of family members in the clinical area because it limited their nursing care. While the nurses in the present study welcomed the presence of family members and parents [12,13]. Other studies have found that nurses view parents as knowing their children better than they do. Even the presence of a parent can reduce the anxiety in the child [11]. 

Nurses agreed that parents should be allowed to care for their sick children and that nurses should be involved only when parents are unable to perform the care. Even though parents were worried about their child’s condition when they could see and care for their child, it helped reduce their anxiety and curtail the uncertainty about their child’s condition. Engaging in care activities made parents happy and satisfied [11,13].

Developing partnerships in care and decision-making was considered a vital element of FCC by the nurses [6]. Nurses supported the family’s individuality. They believed that parents’ and family members' ability to participate in care and decision-making would differ from parent to parent [12,13,6]. Parental presence helped maintain the child’s daily routine, which indirectly promoted the child's developmental needs, nurturing proper growth and development during hospitalization [12-14]. Saudi nurses recognized the necessity of collaboration and information sharing, a view similar to that of nurses in this study [12].

Parents and nurses agreed that sufficient verbal information about their sick child should be provided to parents, and that they should be transparent in communicating the child’s status [10,11]. Some nurses reported that FCC enables nurse-parent relationships, eases collaboration with parents, and empowers families through this trusting nurse-parent relationship and the negotiation of care [10,6]. Whereas Thai nurses considered collaboration the least important aspect of FCC [13].

Nurses noted that maintaining effective communication is the foundation for all involvement and a crucial part of building trust with parents and children. It is helpful to involve parents in all aspects of FCC. Studies have suggested that it is challenging for nurses to practice effective communication when they face a language barrier, findings consistent with the current study, which identified this as an area where nurses need improvement [14,15].

Consistent with other studies, nurses agreed on the importance of FCC. They understood the need for emotional support from family and that familial presence could enhance communication and information sharing. Studies have reported that parental presence can provide comfort to hospitalized children [15,12]. Parents helped nurses receive early notification of the child’s reactions to any medications or treatments and assisted in calming the child. Moreover, nurses reported that parental presence decreased workload; however, they were concerned about parents’ presence during painful procedures [11]. When comparing perceptions and demographics, no differences were found in nurses' views across units. In contrast, less experienced nurses were more aware of the principles of FCC and its philosophy [11,15]. Another study found that both perceptions and practices of FCC were influenced by years of professional experience and clinical area of work [5]. Participants viewed the FCC as contributing to early discharge and better health outcomes for children [15]. Parents and children had also expressed that FCC can promote early recovery [14].

The nurses in the present study agreed that parents were the primary caregivers who fulfilled all the child’s basic needs. They admitted that when parents participated in care activities, they were passively preparing for home care after discharge [11,15,13]. In line with this, parents in the study agreed that their skills in caring for their child were enhanced. The literature suggests that FCC improves parents’ satisfaction, and those involved in their children's care are more self-assured and capable of caring for their sick children at home [5]. It was beneficial for both the child and the parent to be present and involved in their child’s care [10].

The limitations of the study include that a mixed-methods approach could have been applied to determine the perception rate for each component of the FCC. Other methods of data collection, like observation and interviews with parents, would have added value to the data and findings. The study's strengths lie in its inclusion of pediatric nurses working in pediatric surgery, medical, and intensive care settings, which helped to explore and extract nurses’ perceptions. 

## Conclusions

This study demonstrates that pediatric nurses view FCC as a core principle underpinning holistic child health nursing practice. Nurses identified parental and child-related factors as essential foundations for effective FCC implementation. FCC was further perceived as a comprehensive and interactive process involving child-centered, family-centered, and nurse-centered dimensions. The findings underscore the pivotal role of nurses in facilitating collaborative partnerships with families. Strengthening institutional and educational support for FCC may enhance care quality and health outcomes for children and their families.

## Appendices

1. What do you think about the need of family members in the care of children who are admitted in your unit? (Probes:- for parents, for children, for nurses)

2. What do you think about communication and sharing information with children and family in child care? (Probes:- its importance, methods, uses, what type of information should be shared, to what extent we should share the information, whether any challenges will be there)

3. What is your view about involving parents in the care of their children during hospitalisation? (any procedure, daily care, for cooperation from the child)

4. What do you feel about teaching parents the care procedures so that they can take care of their child along with you? (probes:- type of procedures, benefits, demerits)

5. What is your opinion about having policies to involve the parents also in caring their children when they are sick?

6. What is your view about involving parents in decision-making regarding their child’s care and treatment in the hospital?
